# Socio‐economic status and adherence to HIV preventive and therapeutic interventions: exploring the mediating role of food insecurity among men who have sex with men and transgender and non‐binary persons from Brazil

**DOI:** 10.1002/jia2.26432

**Published:** 2025-03-05

**Authors:** Paula M. Luz, Thiago S. Torres, Victor C. Matos, Giovanna G. Costa, Brenda Hoagland, Cristina Pimenta, Marcos Benedetti, Beatriz Grinsztejn, Valdilea G. Veloso

**Affiliations:** ^1^ Instituto Nacional de Infectologia Evandro Chagas Fundação Oswaldo Cruz (INI‐Fiocruz) Rio de Janeiro Brazil; ^2^ Escola Nacional de Saúde Pública Sérgio Arouca Fundação Oswaldo Cruz (ENSP‐Fiocruz) Rio de Janeiro Brazil

**Keywords:** food insecurity, adherence, antiretroviral treatment, pre‐exposure prophylaxis, people living with HIV, Brazil

## Abstract

**Introduction:**

Brazil offers free‐of‐charge antiretroviral therapy (ART) for people living with HIV (PLWH) as well as oral pre‐exposure prophylaxis (PrEP) through its national health system. Adherence to ART and to PrEP is essential to achieving the expected benefits of virologic suppression and prevention of HIV acquisition, respectively. Brazil has experienced worsening social inequalities, exacerbated by the COVID‐19 pandemic, leading to increases in food insecurity especially among vulnerable populations. We explored whether food insecurity mediated the association of socio‐economic status on adherence to ART/PrEP.

**Methods:**

Adult men who have sex with men (MSM) and transgender and non‐binary persons (TGNB) living in Brazil (May−September/2021) voluntarily participated in a cross‐sectional online study advertised on dating apps and social media. Participants living with HIV reporting ART use and participants with HIV‐negative status reporting daily oral PrEP use were eligible for the analysis. Self‐report of ART adherence was measured by the WebAd‐Q instrument (3‐items/past week) plus a visual analogue scale. Self‐report of PrEP adherence was measured by the number of days the person took PrEP in the past week. The 8‐item Brazilian Scale of Food Insecurity (EBIA) was used to measure food insecurity (higher scores indicate more severe food insecurity). Two structural equation models were used to assess the direct and indirect effects of variables on ART adherence among PLWH and on PrEP adherence among people using PrEP.

**Results:**

In total, 1230 PLWH were using ART, and 991 individuals with HIV‐negative status were using daily oral PrEP. The median age of PLWH was 37 years (HIV negative: 34 years), most were cismen (98%). More PLWH reported moderate/severe food insecurity (21.7%; HIV negative: 12.9%). Self‐report of ART adherence (measured by WebAd‐Q, past 7 days) was 55.7% (PrEP adherence: 93.3%). In the two models, socio‐economic status had an effect on adherence that was mediated through food insecurity: higher socio‐economic status was associated with lower food insecurity, and higher food insecurity was associated with lower adherence.

**Conclusions:**

Our findings suggest that the provision of socio‐economic support could help PLWH and people at higher vulnerability to HIV acquisition by improving their adherence to ART or PrEP, and ultimately populations through decreased HIV transmissions.

## 1 INTRODUCTION

Brazil, a middle‐income country with significant socio‐economic challenges, persists as a noteworthy model in public health policies addressing the HIV epidemic. Since 1996, the Brazilian government has provided universal access to antiretroviral therapy (ART), initially based on established immunological criteria, and, since 2013, to all people living with HIV (PLWH) [1]. Starting in late 2017, Brazil provides oral pre‐exposure prophylaxis (PrEP) with the co‐formulation of emtricitabine 200 mg and tenofovir disoproxil fumarate 300 mg (FTC/TDF) to population groups most vulnerable to HIV acquisition with access expanded to populations aged 15 years or older in mid‐2022 [2]. Despite these strides, though the overall HIV prevalence in the country stands at <1%, it is much higher in specific populations, such as gay, bisexual, and other men who have sex with men (MSM), transgender women, sex workers and people who use drugs [3]. Recently, annualized HIV incidence among MSM and transgender and non‐binary persons (TGNB) from Brazil was estimated at 2.62%, with even higher estimates among those aged 18–24 years (3.48%) [4].

Suppression of HIV viral load is indicative of effective HIV management among PLWH, leading to reduced morbidity, improved quality of life and diminished viral transmission risk [5, 6, 7]. Daily adherence to oral ART is critical for sustained virological suppression and improved clinical outcomes. Similarly, the efficacy of daily oral PrEP is highly dependent on adherence, reaching high efficacy in preventing HIV from heterosexual or same‐sex sexual exposure among those who are fully adherent [8, 9, 10].

Similarly to other countries across the globe, in Brazil, approximately 60–70% of PLWH using ART are adherent [11]. ART adherence challenges extend across individual, interpersonal and social determinants of health, including food insecurity [12]. The relationship between food insecurity and HIV operates through multiple pathways and levels—nutritional, mental health, and behavioural at the community, family and individual levels [13]. Insufficient food quality and quantity affects ART absorption, while anxiety about food availability and/or the ability to obtain food may lead to depression, substance use and non‐adherence. Additionally, observational studies have shown an association of food insecurity with sexual behaviours associated with increased risk of HIV acquisition such as inconsistent condom use and engaging in sex work [14, 15, 16]. The stigma associated with food insecurity also plays a role, as it can lead to social isolation and reduced engagement in care [17].

Brazil has experienced worsening social inequalities, exacerbated by the COVID‐19 pandemic, leading to increases in food insecurity especially among vulnerable populations. The assessment of the impact of food insecurity on adherence to ART or PrEP among MSM and TGNB can provide valuable information to tailor public policies that foster social equity. Moreover, when examining additional factors contributing to poor adherence, it is essential to explore specific pathways and mediators as these may provide interesting opportunities for interventions. In this study, we estimated the prevalence of food insecurity among MSM and TGNB using a widely adopted, comprehensive, validated instrument. We also assessed if food insecurity was a mediator of the association of socio‐economic status and ART adherence among PLWH to potentially corroborate and expand prior findings from a single‐centre study conducted in Rio de Janeiro [18]. Lastly, we extend the literature with an evaluation of whether food insecurity was also a mediator of the effect of socio‐economic status and adherence to daily oral PrEP among MSM and TGNB from Brazil.

## 2 METHODS

### 2.1 Study design

A convenience sample of MSM and TGNB chose to voluntarily participate in a cross‐sectional online study advertised in dating apps (Grindr, Hornet and Scruff) and social media (Facebook and Instagram) from May to September 2021. Requests for voluntary study completion were sent through direct message inbox for Hornet, banners for Scruff and Grindr, and boosted posts for Facebook and Instagram, following approaches conducted in prior studies [19, 20]. Only one response per internet protocol address was allowed, if more than one was detected during data management, the oldest (i.e. first) entry was kept. The Instituto Nacional de Infectologia Evandro Chagas (INI‐Fiocruz) institutional review board (CAAE 82021918.0.0000.5262) reviewed and approved this study. As per Brazilian regulations, participants were not reimbursed for participation.

### 2.2 Study population

**Figure 1:**
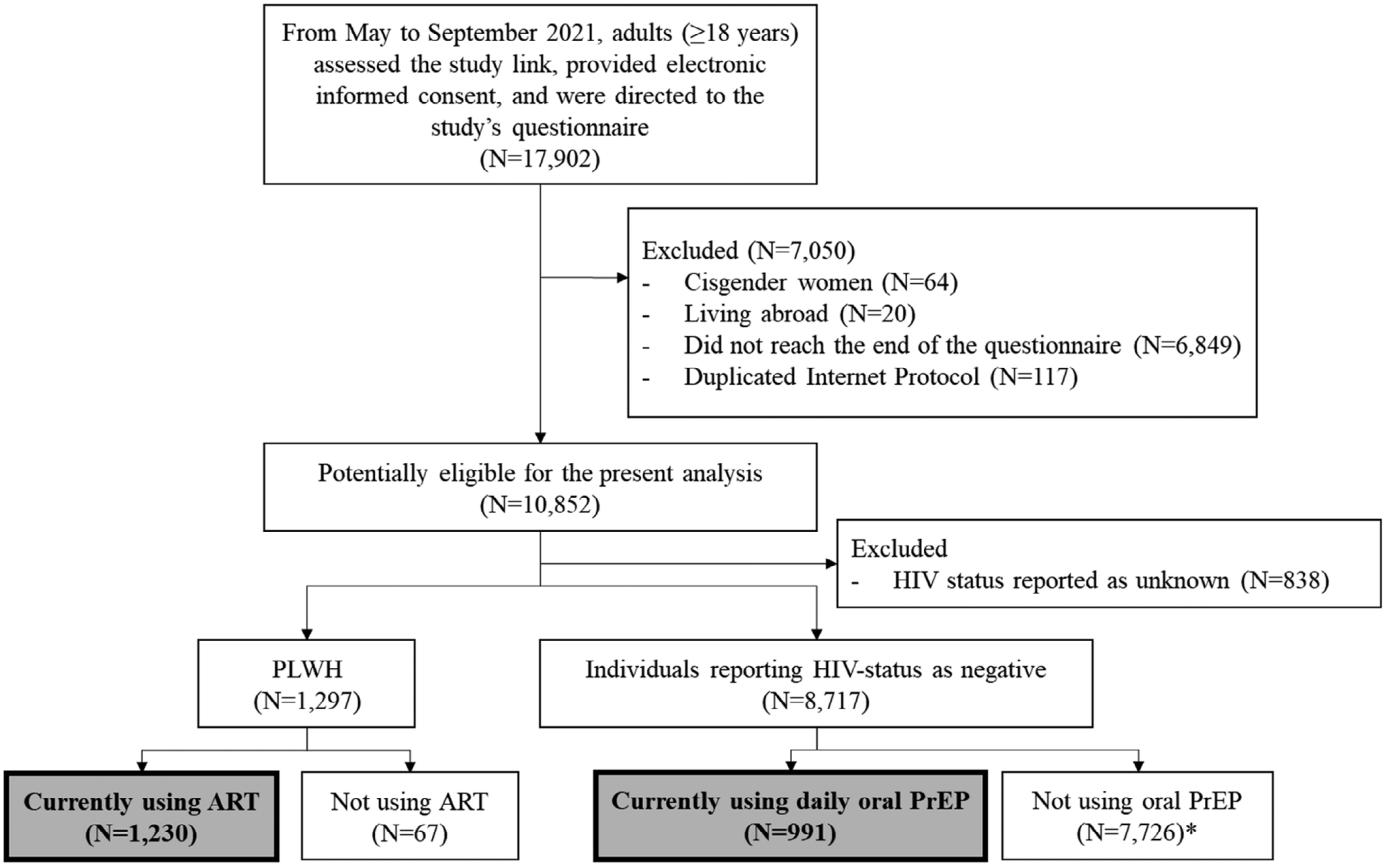
Flow‐chart of study participants, cross‐sectional online study among men who have sex with men and transgender and non‐binary persons from Brazil, May to September 2021. Final, grey‐filled rectangles show participants included in the present analysis. ART, antiretroviral therapy; PLWH, people living with HIV; PrEP, pre‐exposure prophylaxis. *Out of 7726, 7684 individuals reported not using any PrEP modality and 42 individuals reported using other PrEP modalities.

We excluded participants who (1) identified as cisgender women, (2) reported living abroad, (3) did not reach the end of the questionnaire and (4) had duplicated responses based on Internet Protocol (I.P.) address (Figure 1).

### 2.3 Variables

#### 2.3.1 Socio‐demographic characteristics and substance use

Socio‐demographic characteristics included age, race, gender, sexual orientation, education, income and Brazilian state of residence. Race was classified according to the categories defined by the Brazilian Institute of Geography and Statistics: Asian, Black, Indigenous, *Pardo* or multiracial and White. Sexual orientation was collected as gay or homosexual, bisexual, heterosexual and other (e.g. pansexual, asexual or demisexual). The highest level of education attained was categorized as primary, secondary, tertiary and post‐tertiary. Family monthly income was asked in relation to the minimum monthly wage, which was BRL1212 in 2021, equivalent to USD 212. Participants were also asked if, in the past 6 months, they had exchanged sex for money or other resources such as food, housing or gifts. Brazilian state of residence was collected and grouped according to the country's administrative macro‐regions. Substance use in the past 6 months was assessed for: cannabis, cocaine, methamphetamine, poppers and lysergic acid diethylamide (LSD) or other hallucinogenic drugs. Participants were also asked if they had engaged in binge drinking, defined as having more than five drinks in less than 2 hours, in the past 6 months (“Yes,” “No”).

#### 2.3.2 HIV status, sexual behaviour and ART/PrEP use

Participants were asked about the timing of the most recent HIV test and test result which was categorized into “HIV negative,” “PLWH” and “unknown” (had never tested or did not report a test result). Participants reporting HIV‐negative status were presented with four questions (response options) regarding sexual activity in the past 6 months: (1) How many people did you have sex with? (0, 1–5, 6–10, >10); (2) Did you engage in condomless receptive anal sex? (Yes/No); (3) How many times did you have sex per week on average? (≤1, 2, 3–4, 5–6, ≥7); and (4) Did you have a sexually transmitted infection such as syphilis, urethral or rectal gonorrhoea, urethral or rectal chlamydia? (Yes/No). Subsequently, participants were asked if they were aware of PrEP and, if yes, if they were currently using daily oral PrEP. PLWH were asked if they had initiated ART. Those answering affirmatively to ART/PrEP use were subsequently asked about adherence (details below).

#### 2.3.3 Food insecurity

We used the Brazilian Household Food Insecurity Measurement Scale (Escala Brasileira de Medida Domiciliar de Insegurança Alimentar, EBIA), a widely used instrument to measure household food security in Brazil [21]. The full instrument has 14 items: eight that are applicable to an adult, and six exclusively asked in households with persons <18 years of age. Given our expectation that most participants would be young and to decrease instrument length, we included only the eight items applicable to an adult. This approach has been taken in other studies and the psychometric properties of the eight adult items have been assessed and deemed high even for households with children and/or adolescents [22]. In the present, we describe the study population according to proposed cut‐off points (score ≥4 points indicates moderate/severe food insecurity [23]), while considering the items as indicators of the latent variable food insecurity as per the modelling approach described below.

#### 2.3.4 ART/PrEP adherence

ART adherence was measured via the WebAd‐Q instrument, which consists of three questions inquiring on the number of ART doses that were missed or incorrectly taken in the past 7 days. This instrument was developed through interviews and focus groups and was validated against other measures of adherence in the Brazilian context [24]. As per the instrument's design, participants who answered “no” to all three questions were considered with adherence. ART adherence was also assessed with the question “Please mark below the value that corresponds to how much of your antiretroviral medication you took in the past 30 days” to be answered on a visual analogue scale varying from 0% to 100%; individuals who marked 100% were considered with adherence.

PrEP adherence was assessed with one question inquiring on the number of days when PrEP was taken in the past 7 days with the following response options: 7 (all) days, 6 days, 5 days, 4 days, 3 days, 2 days, 1 day and never. Participants who reported taking PrEP 7 days per week were deemed with adherence. Prior work from our group supports these cut‐offs as self‐report of full adherence was highly correlated with protective FTC/TDF blood levels [25, 26].

#### 2.3.5 Theoretical models

**Figure 2:**
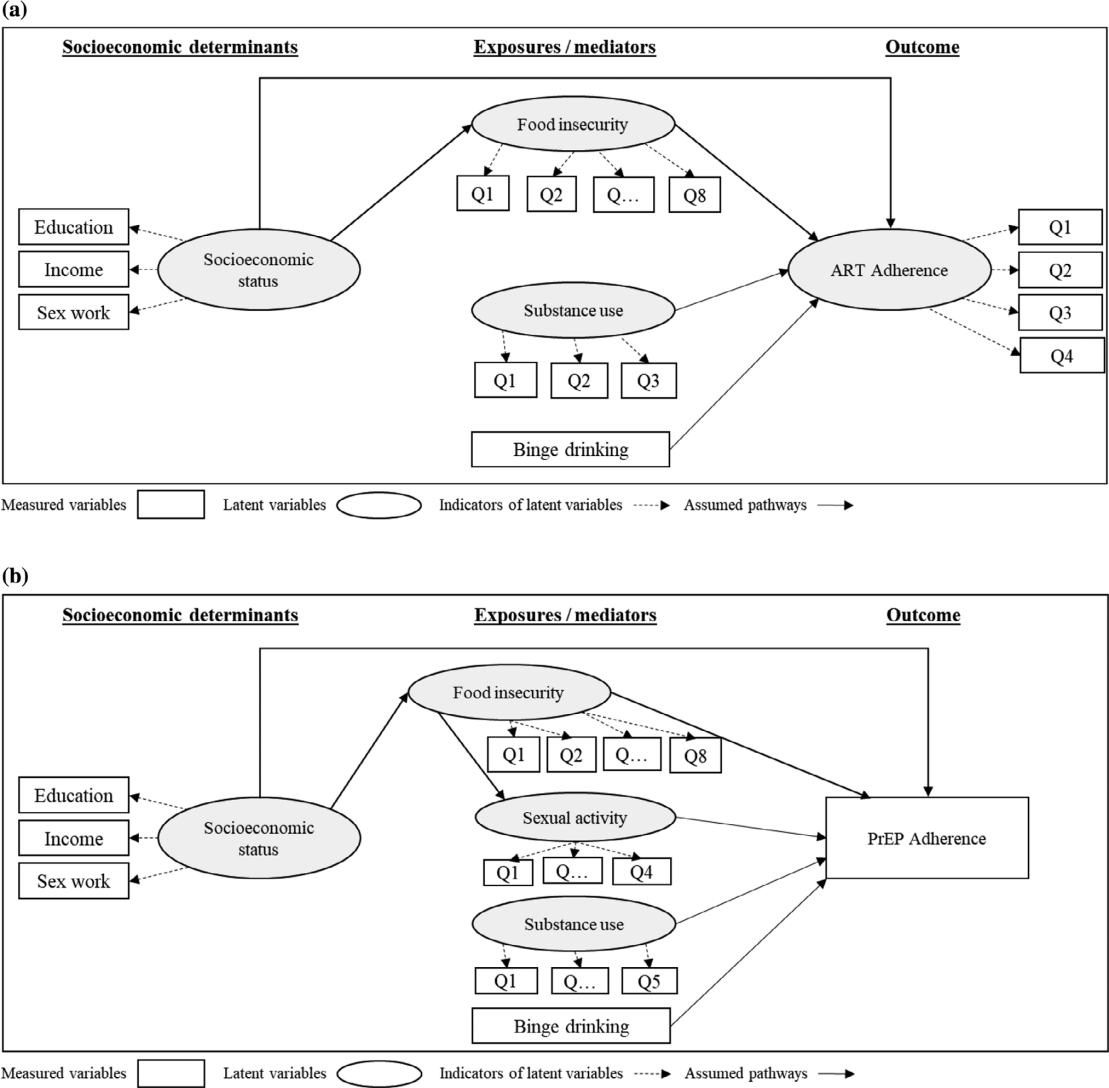
Theoretical model showing the presumed effects of socio‐economic status, food insecurity, substance use and binge drinking on antiretroviral therapy (ART) adherence among people living with HIV (A) and PrEP adherence among participants with HIV‐negative status using daily oral PrEP (B). Socio‐economic status was hypothesized to influence ART/PrEP adherence through direct and indirect (through food insecurity) pathways. Additionally, in (B), a path from food insecurity to sexual activity and a path from sexual activity to PrEP adherence were included.

The two theoretical models one for PLWH using ART and another for participants with HIV‐negative status using PrEP are shown in Figure 2A,B. Having the socio‐ecological model as a frame of reference, we used results from a prior study [18] as well as literature from other low‐ and middle‐income countries to orient our hypotheses about the role of socio‐economic status and of food security on adherence [27, 28, 29, 30, 31, 32]. Socio‐economic status, a more distal predictor, was hypothesized to influence ART/PrEP adherence through a direct path and also indirectly, through food insecurity (Figure 2A,B). Because substance use and binge drinking have been found associated with adherence [11, 18, 33], these variables were included as additional proximal predictors. Additionally, when modelling PrEP adherence (Figure 2B), we included sexual activity as a proximal predictor [34, 35] and hypothesized that food insecurity would be associated with sexual activity, as prior studies have shown [13, 36]. Race was hypothesized to influence socio‐economic status. All structural equations were adjusted for age. Gender or sexual orientation was not included in the model due to small numbers as participants were predominantly (>98%) cisgender MSM.

### 2.4 Statistical analysis

Characteristics of the two population groups, PLWH using ART and participants with HIV‐negative status using PrEP, are provided. Numerical variables were described by their median, interquartile range, and categorical variables by absolute and relative numbers. Differences between none/mild food insecurity and moderate/severe food insecurity among participants using ART and participants using PrEP for categorical and continuous variables were estimated via Pearson's chi‐squared test and Student's *t*‐test, respectively.

Our analytical approach utilized structural equation models (SEM) to simultaneously conduct confirmatory factor analysis and multiple regressions, thereby assessing both the direct and indirect effects of variables on the outcome [37]. Socio‐economic status, food insecurity and substance use were modeled as latent variables (Figure 2A,B). Adherence to ART was also a latent variable in the SEM for PLWH, defined by the three items of the WebAd‐Q questionnaire and the visual analogue scale (Figure 2A). In the SEM for people using PrEP, adherence to PrEP was a measured dichotomous variable (Figure 2B). Standardized estimates with factor loading >0.5 with *p*<0.05 were assumed as an indication that the correlation between the indicator variable and the latent variable was of moderately high magnitude [37]. For SEM, we used a weighted least squares estimator with a diagonal weight matrix and delta parametrization. The amount of missing data was minimal (shown in Table 1) and was handled during the estimation process using pairwise deletion. We used the following goodness of fit statistics (and cut‐offs): (1) the root mean‐square error of approximation (RMSEA, cut‐off<0.06); (2) the comparative fit index and the Tucker Lewis index (CFI/TLI, cut‐off>0.95); and (3) standardized root mean residual (SRMR, cut‐off<0.05) [38, 39]. The direct and indirect effects of the latent and observed variables were assessed in the final model with an effect deemed present when *p*<0.05. R Statistical Software, version 4.02, was used for descriptive analysis, and Mplus software, version 8.10 (Los Angeles, California, USA), for SEM.

**Table 1:** Characteristics of the two study populations, people living with HIV (PLHIV) using antiretroviral treatment (ART) and people with HIV‐negative status using daily oral preexposure prophylaxis (PrEP) stratified by none/mild versus moderate/severe food insecurity as measured by the 8‐item Brazilian Scale of Food Insecurity (EBIA), cross‐sectional online study among men who have sex with men and transgender and non‐binary persons from Brazil, May to September 2021

	PLHIV using ART				HIV‐negative using daily oral PrEP			
	EBIA<4	EBIA≥4^a^	Total	*p*‐value^b^	EBIA<4	EBIA≥4^a^	Total	*p*‐value^b^
	963	267	1230		863	128	991	
**Age (years)**				<0.001				<0.001
Median (IQR)	38 (32,44)	34 (29,41)	37 (31,43)		35 (30,40)	31 (28,38)	34 (29,40)	
**Gender**				0.083				0.221
Cisman	946 (98.2)	257 (96.3)	1203 (97.8)		853 (98.8)	125 (97.7)	978 (98.7)	
Transman	3 (0.3)	0 (0)	3 (0.2)		2 (0.2)	0 (0)	2 (0.2)	
Transwoman	2 (0.2)	1 (0.4)	3 (0.2)		3 (0.3)	0 (0)	3 (0.3)	
Non‐binary	12 (1.2)	9 (3.4)	21 (1.7)		5 (0.6)	3 (2.3)	8 (0.8)	
**Orientation**				0.161				0.073
Gay	879 (91.3)	240 (89.9)	1119 (91)		795 (92.1)	111 (86.7)	906 (91.4)	
Bisexual	73 (7.6)	19 (7.1)	92 (7.5)		58 (6.7)	17 (13.3)	75 (7.6)	
Heterosexual	7 (0.7)	6 (2.2)	13 (1.1)		6 (0.7)	0 (0)	6 (0.6)	
Other	4 (0.4)	2 (0.7)	6 (0.5)		4 (0.5)	0 (0)	4 (0.4)	
**Race**				<0.001				0.001
White	575 (59.7)	114 (42.7)	689 (56)		540 (62.6)	66 (51.6)	606 (61.2)	
Pardo	266 (27.6)	98 (36.7)	364 (29.6)		227 (26.3)	34 (26.6)	261 (26.3)	
Black	104 (10.8)	47 (17.6)	151 (12.3)		83 (9.6)	24 (18.8)	107 (10.8)	
Asian	6 (0.6)	4 (1.5)	10 (0.8)		10 (1.2)	1 (0.8)	11 (1.1)	
Indigenous	12 (1.2)	4 (1.5)	16 (1.3)		3 (0.3)	3 (2.3)	6 (0.6)	
**Education**				<0.001				<0.001
Incomplete primary	9 (0.9)	6 (2.2)	15 (1.2)		1 (0.1)	1 (0.8)	2 (0.2)	
Complete primary	36 (3.7)	24 (9)	60 (4.9)		11 (1.3)	6 (4.7)	17 (1.7)	
Secondary	291 (30.2)	125 (46.8)	416 (33.8)		145 (16.8)	45 (35.2)	190 (19.2)	
Tertiary	366 (38)	73 (27.3)	439 (35.7)		372 (43.1)	49 (38.3)	421 (42.5)	
Post‐tertiary	261 (27.1)	39 (14.6)	300 (24.4)		334 (38.7)	27 (21.1)	361 (36.4)	
**Income (monthly)**				<0.001				<0.001
No income	55 (5.7)	42 (15.7)	97 (7.9)		48 (5.6)	24 (18.8)	72 (7.3)	
Up to 1 mw	56 (5.8)	63 (23.6)	119 (9.7)		31 (3.6)	18 (14.1)	49 (4.9)	
1−2 mw	131 (13.6)	80 (30)	211 (17.2)		78 (9)	35 (27.3)	113 (11.4)	
2−3 mw	126 (13.1)	36 (13.5)	162 (13.2)		128 (14.8)	19 (14.8)	147 (14.8)	
3−4 mw	136 (14.1)	13 (4.9)	149 (12.1)		90 (10.4)	15 (11.7)	105 (10.6)	
4−6 mw	146 (15.2)	18 (6.7)	164 (13.3)		151 (17.5)	11 (8.6)	162 (16.3)	
6−10 mw	162 (16.8)	7 (2.6)	169 (13.7)		151 (17.5)	4 (3.1)	155 (15.6)	
>10 mw	151 (15.7)	8 (3)	159 (12.9)		186 (21.6)	2 (1.6)	188 (19)	
**Transactional sex (past 6 months)^c,d^**				<0.001				<0.001
No	895 (93.3)	209 (79.2)	1104 (90.3)		810 (94.1)	104 (81.9)	914 (92.5)	
Yes	64 (6.7)	55 (20.8)	119 (9.7)		51 (5.9)	23 (18.1)	74 (7.5)	
**Region**				0.227				0.304
North	28 (2.9)	9 (3.4)	37 (3)		32 (3.7)	1 (0.8)	33 (3.3)	
Northeast	84 (8.7)	23 (8.6)	107 (8.7)		71 (8.2)	15 (11.7)	86 (8.7)	
Central‐west	80 (8.3)	12 (4.5)	92 (7.5)		63 (7.3)	9 (7)	72 (7.3)	
Southeast	619 (64.3)	186 (69.7)	805 (65.4)		571 (66.2)	87 (68)	658 (66.4)	
South	152 (15.8)	37 (13.9)	189 (15.4)		126 (14.6)	16 (12.5)	142 (14.3)	
**Substance use (past 6 months)**								
Cannabis	233 (24.2)	71 (26.6)	304 (24.7)	0.422	299 (34.6)	49 (38.3)	348 (35.1)	0.421
Cocaine	117 (12.1)	45 (16.9)	162 (13.2)	0.044	127 (14.7)	29 (22.7)	156 (15.7)	0.021
Methamphetamine	58 (6)	14 (5.2)	72 (5.9)	0.631	85 (9.8)	16 (12.5)	101 (10.2)	0.355
Poppers	20 (2.1)	9 (3.4)	29 (2.4)	0.218	50 (5.8)	3 (2.3)	53 (5.3)	0.105
Hallucinogenic	31 (3.2)	10 (3.7)	41 (3.3)	0.672	56 (6.5)	12 (9.4)	68 (6.9)	0.228
**Binge drinking (past 6 months)**	603 (62.6)	157 (58.8)	760 (61.8)	0.256	536 (62.1)	76 (59.4)	612 (61.8)	0.553
**WebAdQ items (past 7 days)**								
**1. Took medications outside of the hours prescribed**				<0.001				
No	624 (64.8)	115 (43.1)	739 (60.1)					
Yes	329 (34.2)	149 (55.8)	478 (38.9)					
I do not remember	10 (1)	3 (1.1)	13 (1.1)					
**2. Forgot to take medications**				<0.001				
No	823 (85.5)	185 (69.3)	1008 (82)					
Yes	134 (13.9)	80 (30)	214 (17.4)					
I do not remember	6 (0.6)	2 (0.7)	8 (0.7)					
**3. Took less or more pills of medications**				<0.001				
No	898 (93.3)	232 (86.9)	1130 (91.9)					
Yes	60 (6.2)	29 (10.9)	89 (7.2)					
I do not remember	5 (0.5)	6 (2.2)	11 (0.9)					
**ART adherence by WebAdQ^e^**				<0.001				
No	383 (39.8)	166 (62.2)	549 (44.6)					
Yes	580 (60.2)	101 (37.8)	681 (55.4)					
**ART adherence by visual analogue scale (past 30 days)**				<0.001				
<100%	248 (25.8)	100 (37.5)	348 (28.3)					
100%	715 (74.2)	167 (62.5)	882 (71.7)					
**Number of sex partners (past 6 months)^d^**								0.997
None					15 (1.7)	2 (1.6)	17 (1.7)	
1−5					299 (34.7)	44 (34.9)	343 (34.8)	
6−10					179 (20.8)	27 (21.4)	206 (20.9)	
More than 10					368 (42.7)	53 (42.1)	421 (42.7)	
**Condomless receptive anal sex (past 6 months)^d^**								0.065
No					268 (31.1)	37 (29.1)	305 (30.9)	
Yes					593 (68.9)	90 (70.9)	683 (69.1)	
**Number of times had sex per week (past 6 months)**								0.004
1 or less					320 (37.7)	38 (30.2)	358 (36.8)	
Twice					254 (30)	28 (22.2)	282 (29)	
3−4 times					190 (22.4)	37 (29.4)	227 (23.3)	
5−6 times					41 (4.8)	10 (7.9)	51 (5.2)	
7 or more					13 (1.5)	7 (5.6)	20 (2.1)	
Do not know					30 (3.5)	6 (4.8)	36 (3.7)	
**Had STI (past 6 months)**								0.151
No					666 (77.2)	90 (70.3)	756 (76.3)	
Yes					193 (22.4)	37 (28.9)	230 (23.2)	
Do not know					4 (0.5)	1 (0.8)	5 (0.5)	
**PrEP adherence (past 7 days)**								0.089
No					53 (6.1)	13 (10.2)	66 (6.7)	
Yes					810 (93.9)	115 (89.8)	925 (93.3)	

Abbreviations: ART, antiretroviral therapy; IQR, interquartile range; mw, minimum wages; STI, sexually transmitted infection.

^a^ The Brazilian Household Food Insecurity Measurement Scale (Escala Brasileira de Medida Domiciliar de Insegurança Alimentar, EBIA) aims to quantify the presence and severity of food insecurity within households. We used the 8‐items EBIA instrument designed to capture three main aspects of food insecurity: worry about food availability, poor quality of diet due to financial constraints and reduction in quantity of food consumed. A sample question is “In the past 3 months, have you worried that food would run out before there was money to purchase more?” Responses include “Yes,” “No,” “I don't know.” Each affirmative response scores 1 point such that the more questions answered affirmatively, the higher the severity of food insecurity. We used the proposed cut‐off of 4 or more points on the EBIA scale as indicative of moderate to severe food insecurity.

^b^ Differences between none/mild food insecurity (EBIA score <4) and moderate/severe food insecurity (EBIA score ≥4) among PLHIV and HIV‐negative participants using daily oral PrEP for categorical and continuous variables were estimated via Pearson's chi‐squared test and Student's *t*‐test, respectively.

^c^ Defined as sex in exchange for money or other resources such as food, housing or gifts.

^d^ Missing: Ten participants had missing value in the variable transactional sex (seven PLWH and three HIV‐negative participants using daily oral PrEP), four participants had missing value in the variable number of sex partners and three participants had missing value in the variable condomless receptive anal sex.

^e^ The WebAd‐Q instrument consists of three questions inquiring on the number of ART pills that were (1) taken at an incorrect time, (2) not taken or (3) or incorrectly taken in the past 7 days, with possible response options No (0), Yes (1), I don't know (1). Participants who answer “No” to all three questions are deemed adherent as per instrument's design.

## 3 RESULTS

In total, from 14 May to 22 September 2021, 17,902 adults accessed the study link, provided electronic informed consent and were directed to the study's questionnaire, out of which 7050 were excluded (Figure 1). Of the 10,852 potentially eligible participants, 11.9% (1297) reported living with HIV, 80.3% (8717) reported HIV‐negative status and 7.7% (838) reported not knowing their HIV status. A total of 1230 out of 1297 (94.8%) PLWH reported having initiated ART and 991 out of 8717 participants with HIV‐negative status (11.4%) reported using daily oral PrEP; these participants were included in the subsequent analyses (*N* = 2221).

Compared to participants with HIV‐negative status using PrEP (Table 1), PLWH using ART were older (median age 37 years, HIV negative: 34 years), less likely to report as White (PLWH: 56.0%, HIV negative: 61.2%), less likely to have post‐secondary education (PLWH: 60.1%, HIV negative: 78.9%) and more likely to report monthly income of less than one minimum wage or no income (PLWH: 34.8%, HIV negative: 23.6%). The use of substances was more frequently reported by participants using PrEP except for binge drinking. Moderate/severe food insecurity was reported by 21.7% of participants using ART and 12.9% of participants using PrEP. Irrespective of the population group, those reporting moderate/severe food insecurity were younger, with less education and had lower incomes.

Among PLWH, adherence to ART as per the WebAd‐Q instrument was estimated at 55%, whereas through the visual analogue scale, it reached 71.7% of participants. Among participants using oral PrEP, reported adherence was 93.3%. Among PLWH, 60.2% of those without moderate/severe food insecurity were adherent compared to 37.8% of participants with moderate/severe food insecurity (by WebAd‐Q, similar results with the slider question). Among participants using oral PrEP: 93.9% of participants without moderate/severe food insecurity were adherent compared to 89.8% of participants with moderate/severe food insecurity.

### 3.1 SEM model for PLWH using ART: predictors of ART adherence

**Figure 3:**
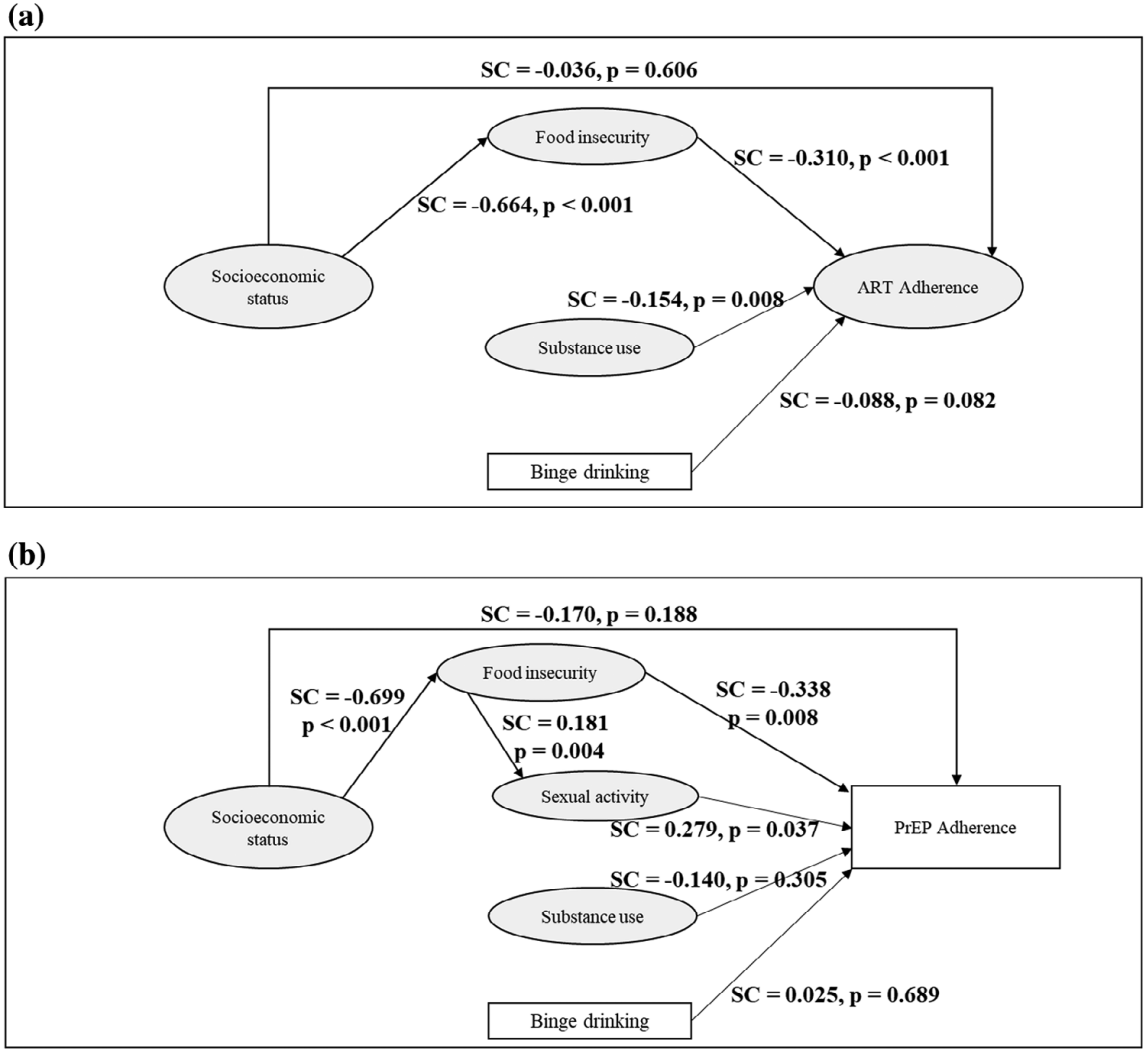
Structural equation models’ standardized coefficients (SC) and *p*‐values (*p*) for paths from latent (ellipse) and measured (rectangle) variables to adherence among people living with HIV using antiretroviral therapy (ART) (A) and participants with HIV‐negative status using daily oral PrEP (B), cross‐sectional online study among men who have sex with men and transgender and non‐binary persons from Brazil, May to September 2021.

Goodness of fit statistics of the proposed model met all established cut‐offs indicative of good fit: RMSEA = 0.022 (90% CI 0.017−0.027), CFI = 0.996, TLI = 0.996, SRMR = 0.054. Effect indicators of latent variables showed good convergent validity, with all standardized factor loadings >0.5 (Table 2). The results showed that socio‐economic status had an indirect effect on ART adherence through food insecurity: higher socio‐economic status was associated with less food insecurity (SC = −0.664, *p*<0.001), and more food insecurity was associated with lower ART adherence (SC = −0.310, *p*<0.001) (Figure 3A and Table 3). Substance use was also associated with lower ART adherence (SC = −0.154, *p* = 0.008) as was binge drinking (SC = −0.088, *p* = 0.082).

**Table 2:** Factor loadings for the measurement models including the latent constructs: socio‐economic status, food insecurity as measured by the 8‐item Brazilian Scale of Food Insecurity (EBIA), substance use, sexual behaviour (only individuals with HIV‐negative status using daily oral PrEP) and antiretroviral treatment (ART) adherence (only people living with HIV), cross‐sectional online study among men who have sex with men and transgender and non‐binary persons from Brazil, May to September 2021

	S.C.	S.E.	Est./S.E.	*p*‐value
**PLHIV using ART**				
**Socio‐economic status**				
Income	0.837	0.031	26.868	<0.001
Education	0.532	0.031	17.032	<0.001
Transactional sex	−0.511	0.064	−7.938	<0.001
**Food insecurity**				
Item 1: worried about running out of food	0.872	0.016	54.422	<0.001
Item 2: ran out of food	0.930	0.011	81.202	<0.001
Item 3: no money for healthy diet	0.935	0.011	88.994	<0.001
Item 4: skipped meal	0.959	0.008	115.195	<0.001
Item 5: cut meal size	0.966	0.008	126.965	<0.001
Item 6: felt hungry	0.956	0.010	99.492	<0.001
Item 7: lost weight	0.927	0.013	69.150	<0.001
Item 8: a day without food/just one meal	0.924	0.016	58.839	<0.001
**Substance use**				
Cocaine	0.983	0.057	17.112	<0.001
Cannabis	0.539	0.052	10.420	<0.001
Methamphetamine	0.852	0.053	16.203	<0.001
**ART adherence**				
Item 1: took meds at wrong time	0.733	0.036	20.642	<0.001
Item 2: did not take prescribed meds	0.976	0.031	31.533	<0.001
Item 3: took meds incorrectly	0.691	0.044	15.634	<0.001
Visual analogue scale	0.778	0.033	23.347	<0.001
**Participants with HIV‐negative status using daily oral PrEP**				
**Socio‐economic status**				
Income	0.763	0.046	16.916	<0.001
Education	0.457	0.045	10.204	<0.001
Transactional sex	−0.520	0.079	−6.359	<0.001
**Food insecurity**				
Item 1: worried about running out of food	0.858	0.021	40.935	<0.001
Item 2: ran out of food	0.875	0.022	39.621	<0.001
Item 3: no money for healthy diet	0.903	0.016	56.242	<0.001
Item 4: skipped meal	0.962	0.011	89.012	<0.001
Item 5: cut meal size	0.980	0.009	110.588	<0.001
Item 6: felt hungry	0.939	0.015	61.05	<0.001
Item 7: lost weight	0.937	0.017	54.287	<0.001
Item 8: a day without food/just one meal	0.909	0.025	36.839	<0.001
**Substance use**				
Cocaine	0.839	0.031	26.755	<0.001
Cannabis	0.529	0.048	11.136	<0.001
Methamphetamine	0.932	0.028	33.533	<0.001
Poppers	0.859	0.038	22.697	<0.001
LSD	0.626	0.058	10.877	<0.001
**Sexual behaviour**				
Number of partners	0.611	0.062	11.356	<0.001
Condomless receptive anal sex	0.314	0.059	5.728	<0.001
Number of sex acts/week	0.412	0.044	7.720	<0.001
Sexually transmitted infections	0.567	0.060	8.769	<0.001

**Table 3:** Estimates from the final structural equation models quantifying the direct and indirect pathways between socio‐economic status, food insecurity as measured by the 8‐item Brazilian Scale of Food Insecurity (EBIA), binge drinking, and substance use on antiretroviral treatment (ART) adherence among people living with HIV (PLWH) and daily oral PrEP adherence among individuals with HIV‐negative status (this model also includes sexual behaviour), cross‐sectional online study among men who have sex with men and transgender and non‐binary persons from Brazil, May to September 2021

	S.C.	S.E.	Est./S.E.	*p*‐value
**PLWH using ART**				
**Effects on ART adherence**				
Of SES				
Direct pathway	−0.036	0.069	−0.516	0.606
Indirect through food insecurity	0.206	0.047	4.401	<0.001
Of other variables				
Food insecurity	−0.310	0.068	−4.563	<0.001
Substance use	−0.154	0.058	−2.646	0.008
Binge drinking	−0.088	0.051	−1.739	0.082
**Participants with HIV‐negative status using daily oral PrEP**				
**Effects on PrEP adherence**				
Of SES				
Direct pathway	−0.170	0.130	−1.316	0.188
Indirect through food insecurity	0.212	0.104	2.036	0.042
Indirect through food insecurity and sexual behaviour	−0.032	0.022	−1.424	0.154
Of other variables				
Food insecurity	−0.338	0.128	−2.649	0.008
Substance use	−0.140	0.136	−1.025	0.305
Binge drinking	0.025	0.064	0.400	0.689
Sexual behaviour	0.279	0.134	2.087	0.037
**Effect on sexual behaviour**				
Of food insecurity	0.181	0.063	2.872	0.004

Abbreviations: *p*‐value, two tailed *p*‐value; SC, standardized coefficient; SE, standard error; SES, socio‐economic status.

### 3.2 SEM model for participants with HIV‐negative status using PrEP: predictors of PrEP adherence

Goodness of fit statistics of the proposed model met established cut‐offs indicative of good fit except for SRMR: RMSEA = 0.037 (90% CI 0.033−0.041), CFI = 0.977, TLI = 0.974, SRMR = 0.095. Effect indicators of the latent variables food insecurity and substance use showed good convergent validity (factor loadings >0.5, Table 2). For the latent variable socio‐economic status, standardized loading for education level was slightly below the established cut‐off (SC = 0.457, *p*<0.001). For the latent variable sexual behaviour, standardized loadings were <0.5 for the number of sex acts per week (SC = 0.412, *p*<0.001) and condomless receptive anal sex (SC = 0.314, *p*<0.001). Results showed that socio‐economic status had an indirect effect on PrEP adherence through food insecurity: higher socio‐economic status was associated with lower food insecurity (SC = −0.699, *p*<0.001), and higher food insecurity was associated with lower PrEP adherence (SC = −0.338, *p* = 0.008) (Figure 3B and Table 3). Higher food insecurity was associated with higher sexual activity (SC = 0.181, *p* = 0.004). Higher sexual activity was associated with higher PrEP adherence (SC = 0.279, *p* = 0.037).

## 4 DISCUSSION

Between May and September 2021, we utilized online recruitment strategies via dating apps and social media to collect data from over 1200 MSM and TGNB persons living with HIV and on ART, as well as nearly 1000 MSM and TGNB persons with HIV‐negative status using daily oral PrEP in Brazil. We estimated the prevalence of food insecurity using a validated instrument and found that 22% of PLWH experienced moderate to severe food insecurity, a prevalence nearly double that of participants using daily oral PrEP (12%). Furthermore, we found that food insecurity was a mediator of the effect of socio‐economic status on adherence to ART and PrEP.

Since our population groups were engaged simultaneously using identical strategies, the contrasting prevalence of food insecurity likely reflects their underlying vulnerabilities. Compared to participants using PrEP, PLWH using ART were older, more likely to report Black or *Pardo* race, less likely to have post‐secondary education and more likely to report lower monthly income. These findings reflect the impact that socio‐economic vulnerability has on HIV acquisition in Brazil [40]. When considering food insecurity in particular, research has shown an association between food insecurity and sexual behaviours with increased risk of HIV exposure in populations not living with HIV [41, 42] and among PLWH [43]. Experiencing food insecurity and other forms of social deprivation may force individuals towards survival strategies that may involve exchanging sex for money or basic needs like food (i.e. transactional sex). Moreover, experiencing food insecurity is physically and psychologically very stressful to the organism, leading to the depletion of mental resources and decreasing an individual's decision‐making capacity for healthy options, including negotiating safe sex practices. Data from population‐based surveys conducted in six sub‐Saharan African countries corroborate these hypotheses by showing that food insecurity contributed to transactional and condomless sex despite knowledge of and desire for safer sex practices [44]. Our results showed that among participants using daily oral PrEP, higher levels of food insecurity were associated with increased sexual activity, corroborating the findings from the literature.

Results from recent nationally representative surveys of the Brazilian population conducted in 2018, 2020 and 2021/2022 have indicated that moderate/severe food insecurity has increased in the past 6 years [45]. The latter survey reported a prevalence of moderate/severe food insecurity among Brazilians of 30.1%. It is challenging to compare our results to national surveys because national surveys include children, adolescents and cisgender women, which are, by design, populations not included in the present study. Moreover, the recruitment strategies used in the present study favoured the two richest macro‐regions of the country (Southeast and South, Table 1), as well as the inclusion of participants with higher education and income thus decreasing the prevalence of food insecurity. We found only one study that estimated food insecurity among sexual and gender minorities from Brazil, an online study including 112 TGNB, conducted between October and December of 2020 [46]. This study showed even more alarming results, with 20.2% of participants reporting severe food insecurity (EBIA score ≥6); equivalent estimates for our population groups are 13.7% of PLWH using ART and 6.8% of participants with HIV‐negative status using daily oral PrEP.

Our results showed that food insecurity was a mediator of the pathway from socio‐economic status to adherence to ART and PrEP. Participants reporting lower socio‐economic status were more likely to report food insecurity and experiencing food insecurity was associated with lower adherence. Thus, we add to the growing body of knowledge that has shown how food insecurity represents an important barrier to ART adherence and the potential benefits of food assistance programmes in supporting ART adherence [12]. Accordingly, our results support the call for immediate, actionable interventions that could address access to food and long‐term structural interventions to reduce socio‐economic vulnerabilities. Immediate, actionable interventions could include access to food banks, provision of food baskets or food vouchers. The distribution of food stamps has been carried out by the Supplemental Nutrition Assistance Program, a large programme in the United States aiming to reduce food insecurity, with longitudinal results (2003−2010) showing that recipients of the programme had improved dietary quality and better weight status [47]. Long‐term structural interventions may include increasing the minimum wage or providing financial assistance to low‐income households. Results from a large safety‐net programme in Ethiopia using a randomized sampling of 188 households (including recipients and non‐recipients) indicated that the transfers (cash, food or a combination) decreased food insecurity [48]. Similarly, a longitudinal study (2011−2014) from a northeastern city of Brazil, using a randomized sampling of 326 households and the EBIA scale to measure food insecurity, showed that households receiving less cash transfers (*Bolsa Família* programme) during follow‐up were at increased risk of food insecurity [49].

Our study has limitations. As a cross‐sectional survey, we highlight that the proposed relations between variables do not presuppose causality. Additionally, temporality between exposure and mediator variables and outcomes cannot be guaranteed in any cross‐sectional study. To improve data quality, we used simple, clear and concise language, pre‐tested the questionnaires on different platforms and limited the survey length to ∼15 minutes. We also removed participants who did not reach the end of the questionnaire and duplicated I.P. addresses. However, we did not employ other means of ensuring data quality such as attention checks. As participants’ responses were self‐reported, they may have been subject to social desirability bias. PrEP adherence in our sample was high and although there is literature suggesting that self‐reported adherence may be overestimated, our neutral assessment may have minimized such bias. Moreover, studies have reported high PrEP adherence among individuals engaged and motivated in the programme [50], and prior work from our group has shown that self‐report adherence can discriminate people who use PrEP that have and do not have protective drug levels [25]. Most of our sample (∼65%) was from southeast Brazil limiting the generalizability of our descriptive findings to all regions of the country. Given the required access to a device compatible with geosocial networking applications and internet connection for study participation, our sample might represent individuals of higher socio‐economic status, when compared to a broader population of Brazil. In the present study, to minimize participant burden, we did not ask PLWH about their sexual behaviour. It may be interesting to explore how sexual behaviour relates to ART adherence in future studies.

## 5 CONCLUSIONS

Brazil's progress in managing the HIV epidemic has been marked by commendable policies, but persistent social and structural challenges remain. Presently, to fully address the HIV epidemic requires an understanding and acknowledging of the social determinants shaping the health outcomes of MSM and TGNB living with HIV and vulnerable to its acquisition. Our findings indicate that MSM and TGNB in Brazil living with HIV or at risk of acquiring it were less likely to adhere to their medications if they experienced food insecurity. This suggests that interventions aimed at improving adherence to ART and PrEP should address food insecurity as a means to alleviate the impact of poverty. Provision of food or food vouchers could directly help PLWH by improving their adherence. Moreover, individuals at higher vulnerability to HIV acquisition who have initiated PrEP and have lower socio‐economic status could also benefit from food support which would improve their adherence. These interventions could ultimately benefit broader populations through decreased HIV transmissions.

## COMPETING INTERESTS

The authors declare no competing interests.

## AUTHORS’ CONTRIBUTIONS

PML and TST proposed the research question and conceived the analysis. PML, TST, BH, CP, MB, BG and VGV conceived and implemented the study. PML, TST and VCM analysed the data and generated the results. GGC, BH, CP, BG and VGV provided guidance on results interpretation. PML, TST and VCM reviewed the literature and drafted the manuscript. All authors critically revised the manuscript for important intellectual content and approved the final version of the manuscript.

## FUNDING

This project was made possible thanks to Unitaid's funding and support. Unitaid accelerates access to innovative health products and lays the foundations for their scale‐up by countries and partners. Unitaid is a hosted partnership of the World Health Organization. PML was supported by the National Council of Technological and Scientific Development (CNPq; #316401/ 2021–8) and Carlos Chagas Filho Foundation for Research Support in the State of Rio de Janeiro (FAPERJ; #E‐26/201.133/2021). TST was supported by CNPq (#402916/2021‐2 and #311871/2021‐6) and FAPERJ (#E‐26/211.577/2021 and #E‐26/201.270/2022). BG was supported by CNPq (#313265/2023‐2) and FAPERJ (#E.26/200.946/2022). We thank the Ministry of Health of Brazil for its support.

## Data Availability

A complete de‐identified dataset sufficient to reproduce the study findings will be made available upon request to the corresponding author, following approval of a concept sheet summarizing the analyses to be done.
